# Ghrelin’s potential as a therapeutic target for chronic inflammatory diseases: evidence from human endometrial stromal cells

**DOI:** 10.3389/fendo.2025.1587490

**Published:** 2025-09-01

**Authors:** Wenhui Dong, Hongkai Mu, Fan Jia, Yingying Wei, JingJing Lv, Shizhao Zhou, Shiping Yu, Tingting Liang

**Affiliations:** ^1^ Medical Imaging Department, Shanxi Medical University, Taiyuan, China; ^2^ Department of Interventional Therapy, Second Hospital of Shanxi Medical University, Taiyuan, China; ^3^ Department of Interventional Therapy, The First Hospital of Shanxi Medical University, Taiyuan, China; ^4^ Department of Interventional Therapy, Shanxi Province Cancer Hospital/ Shanxi Hospital Affiliated to Cancer Hospital, Chinese Academy of Medical Sciences/ Cancer Hospital Affiliated to Shanxi Medical University, Taiyuan, China; ^5^ Obstetrics and Gynecology Department, Lishi District People’s Hospital of Lvliang City, Lvliang, Shanxi, China; ^6^ Obstetrics and Gynecology Department, Second Hospital of Shanxi Medical University, Taiyuan, China

**Keywords:** Ghrelin, inflammation, cytokines, endometrial stromal cells, anti-inflammatory, chronic inflammatory diseases

## Abstract

**Background:**

Ghrelin, a peptide composed of 28 amino acids, is recognized for its role in regulating appetite and energy balance. Recently, it has also been identified as an immunomodulator that could significantly influence immune responses in chronic inflammatory conditions. The role of ghrelin on cell viability and cytokine expression is presented here for human endometrial stromal (hEM15A) cells, with attention to the way this peptide could modulate inflammation.

**Methods:**

In this study, the hEM15A cells were cultured and treated with Ghrelin at concentrations ranging from 1 μM to 1000 μM. Cell viability was assessed using the Cell Counting Kit-8 (CCK-8) assay. Levels of the cytokines TNF-α, IL-6, and IL-10 were measured by ELISA, and the expression of the Ghrelin receptor was confirmed through Western blot (WB) analysis.

**Results:**

The results demonstrated successful expression of the Ghrelin receptor (GHSR) in hEM15A cells. Analysis of cell viability indicated that Ghrelin positively affected cell proliferation, particularly at higher concentrations. ELISA results showed a significant decrease in pro-inflammatory cytokines TNF-α and IL-6, coupled with a notable increase in the anti-inflammatory cytokine IL-10, in a dose-dependent manner.

**Conclusion:**

Ghrelin can exert its effects through its receptor GHSR. Meanwhile, Ghrelin stimulates cell growth without causing decrease in viability; it has cell protective effect by regulating inflammation at the molecular level by balancing the release of some key pro-inflammatory cytokines. This study discovered and validated the anti-inflammatory effect of Ghrelin in patients with endometriosis. Thus, the data presented open a potential use of Ghrelin as therapy for chronic inflammation-related disorders as endometriosis.

## 1 Introduction

Ghrelin, primarily secreted by the stomach (1) and, to a lesser extent, by other tissues, acts as an endogenous agonist for the growth hormone secretagogue receptor (GHSR), a G-protein-coupled receptor widely expressed in various tissues, including the brain (2), pancreas (3), and reproductive organs. Beyond its well-established roles in regulating appetite and energy balance, ghrelin has been identified as a key modulator of growth hormone secretion (4), gastrointestinal motility (5), glucose and lipid metabolism, and numerous other physiological processes (6). Emerging evidence suggests that ghrelin exerts significant anti-inflammatory effects, positioning it as a promising pharmacological candidate for managing inflammatory diseases (7).

Ghrelin has been shown to influence immune responses in chronic inflammatory conditions, including endometriosis (8), rheumatoid arthritis (RA) (9), and inflammatory bowel diseases (IBD) (10). The immune response during inflammation involves the secretion of pro-inflammatory cytokines, activation of immune cells, and tissue destruction (11). Chronic inflammation is a major contributor to the pathogenesis of various diseases, particularly those affecting the reproductive system (12). For example, in the endometrium, key cytokines such as tumor necrosis factor-alpha (TNF-α), interleukin-6 (IL-6), and interleukin-10 (IL-10) play pivotal roles in inflammation. TNF-α and IL-6 are recognized as pro-inflammatory cytokines (13), whereas IL-10 functions as an anti-inflammatory cytokine that mitigates tissue damage and promotes healing (14). Maintaining a delicate balance among these cytokines is essential for preserving physiological homeostasis and preventing pathological progression.

Recent studies have highlighted ghrelin’s anti-inflammatory properties in various models of inflammation, demonstrating its ability to reduce pro-inflammatory cytokine levels (15, 16). Thus, ghrelin is not only a metabolic regulator but also an immunomodulator, making it an attractive target for therapeutic interventions in inflammatory diseases (17, 18).

The human endometrium is particularly susceptible to inflammatory reactions. Dysregulated immune responses in the endometrium can lead to disorders such as endometriosis, which is characterized by chronic inflammation and aberrant tissue proliferation (19). The hEM15A cell line serves as a valuable model for studying the effects of ghrelin on endometrial inflammation. Investigating ghrelin’s role in modulating immune responses in endometrial cells, including its effects on cytokine production and interactions with immune cells, may provide novel insights into its therapeutic potential for reproductive disorders (20).

This study aims to evaluate the effects of ghrelin on hEM15A cell viability and cytokine production, specifically focusing on its role in modulating inflammatory responses. By examining ghrelin’s interactions with GHSR and associated downstream signaling pathways, this research seeks to elucidate its protective effects against endometrial inflammation. Additionally, the study will explore ghrelin’s dose-dependent effects on the secretion of TNF-α, IL-6, and IL-10 to identify potential pharmacological strategies for managing endometrial-related inflammatory disorders.

## 2 Materials and methods

### 2.1 Reagents

**Figure 1:**
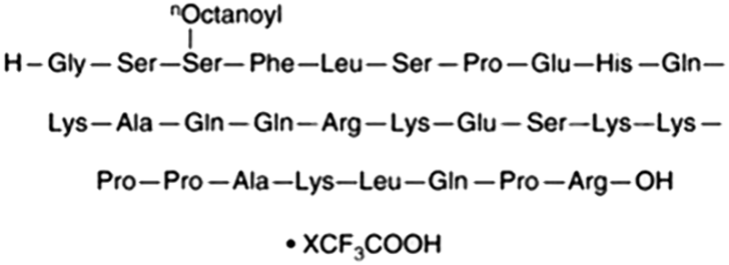
Chemical structures of Ghrelin.

Ghrelin (purity > 98%; **Figure 1**) was obtained from ChemeGen Co., Ltd. (Shanghai, China). The CCK-8 reagent was obtained from YOBIBlO Co., Ltd. (Shanghai, China). ELISA kits for TNF-α, IL-6, and IL-10 were sourced from Beijing Solarbio Science & Technology Co., Ltd. (Beijing, China). WB detection kits, beta-actin polyclonal antibody, and horseradish peroxidase (HRP)-conjugated secondary antibodies were acquired from Elabscience Biotechnology Co., Ltd. (Wuhan, China). GHSR Rabbit pAb were sourced from Zen-Bioscience Co., Ltd. (Chengdu, China).

### 2.2 Cell culture

The hEM15A cells obtained from the China Center for Type Culture Collection (CCTCC, Wuhan, China), and provided with authentication testing reports. The hEM15A cells were cultured in Dulbecco’s Modified Eagle Medium/F-12 (DMEM/F-12, Zhongqiao Xinzhou, Shanghai, China) enriched with 10% fetal bovine serum (FBS; Cellmax, SA211, Beijing, China). The cells were maintained at 37 °C in a humidified atmosphere containing 5% CO_2_ for 24 hours. Cells were stained with Hoechst 33258 and visualized under fluorescence microscopy to confirm the absence of Mycoplasma. Following this, cells were trypsinized for 2 minutes, resuspended in fresh DMEM/F-12 with 10% FBS, and incubated for an additional 24 hours. Ghrelin was then administered in varying concentrations (1000 μM, 100 μM, 10 μM, 1 μM) for 12 hours. A PBS-treated group acted as the negative control. Both culture supernatants and cells were collected for subsequent analyses.

### 2.3 CCK-8 assay

Cell viability was evaluated using the CCK-8 assay. hEM15A cells were plated at 1×10^5^ cells per well in 96-well plates and incubated for 24 hours. The cells were exposed to different concentrations of Ghrelin (1000 μM, 100 μM, 10 μM, 1 μM) for 24 hours, followed by the addition of 10 μL of CCK-8 reagent to each well. Make 5 repeated wells for each concentration. After a further 2-hour incubation, optical density (OD) was measured at 450 nm using a Multiskan FC microplate reader (MA, USA). Cell viability percentage was determined using the formula: (OD _treatment_–OD _blank_)/(OD _control_–OD _blank_) × 100%.

### 2.4 Cytokine assay by ELISA

Cytokine levels for IL-10, TNF-α, and IL-6 were quantified using ELISA kits, under the same experimental conditions as previously described. Supernatants from hEM15A cells were used for cytokine measurements following the protocols provided with ELISA kits from Beijing Solarbio Science & Technology Co., Ltd. (Beijing, China). A PBS-treated group served as the negative control. Repeat the experiment three times.

### 2.5 Western blot

Proteins were extracted from tissues/cells using RIPA buffer supplemented with protease/phosphatase inhibitors, and concentrations were determined via BCA assay. Equal amounts of protein (50 μg) were separated by SDS-PAGE (10% gels) and transferred to PVDF membranes. After blocking with 5% non-fat milk in TBST, membranes were incubated overnight at 4 °C with primary antibodies (1:1000), followed by HRP-conjugated secondary antibodies (1:5000, 1 h at RT). Signals were detected using ECL substrate and quantified with ImageJ, normalized to β-actin as loading controls.

### 2.6 Statistical analysis

Data analysis was performed using SPSS 23.0 and GraphPad Prism 8.0, with results presented as mean ± standard deviation (SD). Group differences were evaluated using a t-test or one-way analysis of variance (ANOVA), followed by Bonferroni *post hoc* tests as needed. Pearson correlation analysis was applied to explore the association between cytokine levels and GHSR expression. A p-value of <0.05 was deemed statistically significant.

## 3 Results

### 3.1 GHSR expression in hEM15A cells

**Figure 2:**
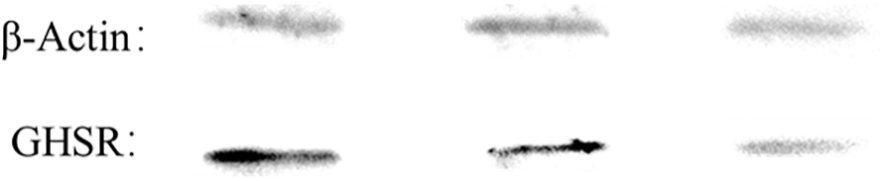
Expression of GHSR in hEM15A cells, analyzed by WB.

To investigate the potential protective effect of Ghrelin on endometrial stromal cell inflammation and its associated signaling mechanisms, the expression of the GHSR was first confirmed in hEM15A cells. WB analysis revealed a significant expression of GHSR in these cells, validating its suitability for subsequent studies involving Ghrelin (**Figure 2**).

### 3.2 Effect of Ghrelin on cell viability

**Figure 3:**
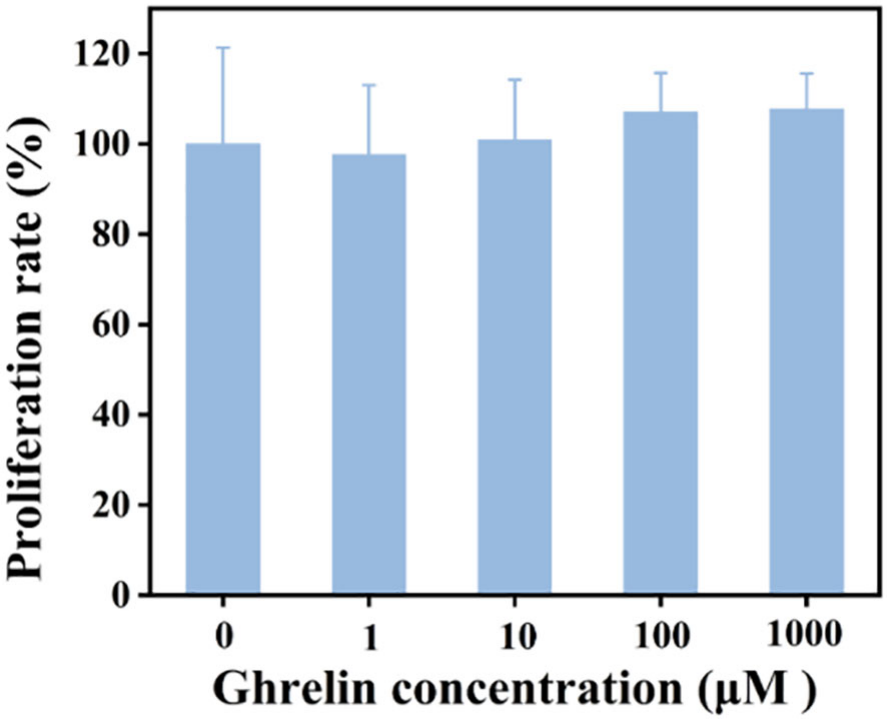
Comparison of cell survival rates for hEM15A cells incubated with various concentrations of Ghrelin over 24 hours (n = 5).

The CCK-8 assay was employed to assess the impact of Ghrelin on the viability of hEM15A cells. Cells were exposed to varying Ghrelin concentrations for 24 hours. As depicted in **Figure 3**, cell viability remained over 80% across all concentrations, including the highest at 1000 μM, suggesting that Ghrelin does not adversely affect cell survival. It is of interest that cell viability exceeded 100% at higher Ghrelin concentrations, which may point toward a proliferative effect of Ghrelin. This makes it probable that Ghrelin promotes proliferation, though this is an interesting phenomenon that needs further investigation.

### 3.3 Effect of Ghrelin on cytokine expression

**Figure 4:**
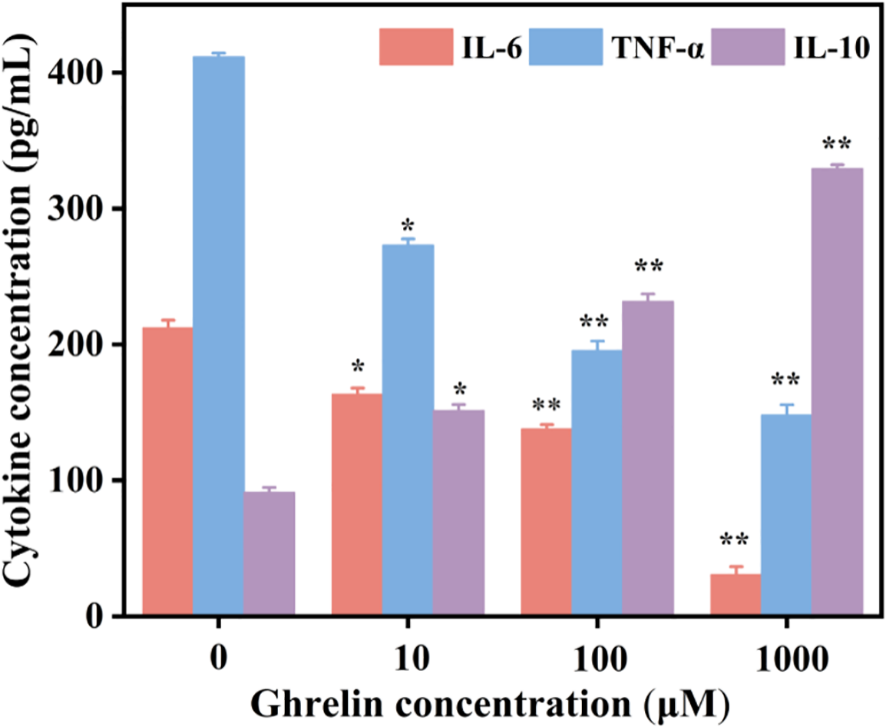
The concentrations of IL-10, TNF-α, and IL-6 in supernatants following treatment with different concentrations of Ghrelin were quantified by ELISA (n = 3). Data in panels are presented as mean ± standard deviation. *p<0.05, **p<0.01.

The effect of Ghrelin on inflammatory cytokine production was quantified using ELISA in hEM15A cell supernatants. Post-Ghrelin treatment, there was a significant reduction in the levels TNF-α and IL-6, while IL-10 showed. Compared with the control group, the Ghrelin group with a concentration of 1000 μ M showed a 86% reduction in TNF - α, a 64% reduction in IL-6, and a261% increase in IL-10 (**Figure 4**). Notably, increases in Ghrelin concentration led to a dose-dependent decrease in TNF-α and IL-6 levels, alongside an increase in IL-10 levels, illustrating Ghrelin’s anti-inflammatory action through the suppression of pro-inflammatory cytokines and enhancement of anti-inflammatory cytokine secretion.

In summary, Ghrelin demonstrated no negative effect on cell viability at lower concentrations, while promoting cell proliferation at higher doses. Furthermore, Ghrelin regulated immune responses in hEM15A cells by diminishing TNF-α and IL-6, and augmenting IL-10 production in a concentration-dependent manner. These findings indicate Ghrelin’s potential as a therapeutic agent in conditions requiring immune response modulation.

## 4 Discussion

Ghrelin is a peptide initially recognized for its role in appetite regulation and energy homeostasis. Recently, it has garnered attention as a key mediator of immune functions, particularly in chronic inflammatory disorders (1). This study evaluated the effects of Ghrelin on cell viability and cytokine production using hEM15A cells as a model for endometrial inflammation. The results demonstrate that Ghrelin enhances cell proliferation and exhibits anti-inflammatory properties in hEM15A cells, suggesting its potential utility in treating inflammatory diseases such as endometriosis.

The expression of the Ghrelin receptor (GHSR) in hEM15A cells was confirmed through WB analysis. Previous studies have established the involvement of Ghrelin and GHSR in activating intracellular pathways, including MAPK and NF-κB, which are critical regulators of immune responses. The high expression of GHSR observed in hEM15A cells supports their suitability as a model for investigating Ghrelin’s role in endometrial inflammation. This finding aligns with earlier research reporting GHSR expression in various peripheral tissues, immune cell lineages, and other cell types, highlighting Ghrelin’s broad regulatory influence on immune responses beyond its metabolic functions.

Cell viability was assessed using the CCK-8 assay. The results indicate that Ghrelin does not reduce cell viability at concentrations of 1000 μM, 100 μM, 10 μM, or 1 μM. Notably, at 1000 μM, cell viability exceeded 100%, indicating a proliferative effect. This suggests that Ghrelin promotes the proliferation of endometrial stromal cells, consistent with its known proliferative effects on other cell types, such as adipocytes (21) and pancreatic cells (22). Further research is warranted to elucidate the mechanisms underlying this proliferative response and to determine whether it operates in a universal or cell-specific manner.

The study also investigated Ghrelin’s role in modulating inflammatory cytokine release in hEM15A cells. The findings reveal that Ghrelin significantly reduced the secretion of pro-inflammatory cytokines TNF-α and IL-6 while increasing the production of the anti-inflammatory cytokine IL-10 in a dose-dependent manner. The marked reduction in TNF-α and IL-6 is particularly noteworthy, as these cytokines are central to the pathology of chronic inflammatory diseases such as endometriosis (23), rheumatoid arthritis (RA) (24), and inflammatory bowel disease (IBD) (25). The upregulation of IL-10 further underscores Ghrelin’s immunomodulatory capabilities, as it not only suppresses pro-inflammatory mediators but also promotes anti-inflammatory pathways (26). These results are consistent with prior studies highlighting Ghrelin’s role in immune modulation across various inflammatory models.

The anti-inflammatory effects of Ghrelin may involve GHSR-mediated inhibition of inflammatory cascades, including the downregulation and degradation of NF-κB-related factors and the activation of their antagonistic modulators, particularly MAP kinases (27, 28). These pathways are known to influence both innate and adaptive immune responses, affecting macrophages and T cells, which regulate cytokine production and cellular proliferation (29, 30). Ghrelin’s ability to modulate cytokine profiles in inflammatory contexts positions it as a promising candidate for developing novel therapeutic strategies for inflammatory diseases of the endometrium, such as endometriosis, which is characterized by cytokine imbalances.

These findings suggest that Ghrelin could serve as a therapeutic agent in diseases characterized by chronic inflammation, particularly in reproductive organs. By restoring immune homeostasis and modulating cytokine-induced inflammation, Ghrelin may help reduce disease severity in conditions like endometriosis. Additionally, its proliferative effects at higher concentrations indicate potential applications in tissue regeneration, such as post-surgical recovery or repair of damage caused by chronic inflammation (31). This dual role of Ghrelin in immune modulation and tissue healing underscores its significance as a therapeutic target, extending its potential applications in medical treatment.

Although this study explores the potential immunomodulatory role of ghrelin in endometrial cells, certain limitations warrant consideration. First, the present research relies on *in vitro* experiments using a cell line, which cannot fully replicate the complexity of *in vivo* conditions. Therefore, further studies employing animal models are recommended to investigate the effects of ghrelin under more physiologically relevant conditions. Additionally, further research is necessary to elucidate the precise molecular mechanisms by which ghrelin modulates the NF-κB and MAPK pathways, as well as to identify the optimal therapeutic concentration.

Moreover, the long-term effects of ghrelin on tissue remodeling and immune function require further investigation. Clinical trials are critical to assess whether therapeutic strategies involving ghrelin are effective in managing inflammatory disorders, particularly those associated with reproductive health.

## 5 Conclusion

In conclusion, this study provides the first mechanistic insights into Ghrelin’s pivotal role in modulating inflammatory responses in endometrial cells, promoting anti-inflammatory cytokine secretion while repressing pro-inflammatory mediators. These results bolster the potential of Ghrelin as an effective therapeutic agent against chronic inflammatory diseases impacting the endometrium and other tissues. Future studies should investigate Ghrelin’s effects in animal models of endometriosis and explore optimal dosing strategies that balance its anti-inflammatory and proliferative effects.

## Data Availability

The datasets presented in this article are not readily available. The findings of this study are derived from *in vitro* experiments using the immortalized human endometrial stromal cell line hEM15A. While this model provides a controlled system for mechanistic investigations, the results may not fully replicate the complexity of *in vivo* endometrial tissue microenvironments or patient-specific variations. Additionally, the cytokine analysis was limited to TNF-α, IL-6, and IL-10; other inflammatory mediators or signaling pathways potentially influenced by Ghrelin were not explored. Dose-dependent effects were tested within a predefined concentration range, and extrapolation to physiological or therapeutic doses requires further validation. Requests to access the datasets should be directed to 13835184774@163.com.
